# Synergistic innovation in organ-on-a-chip and organoid technologies: reshaping the future of disease modeling, drug development, and precision medicine

**DOI:** 10.1093/procel/pwaf058

**Published:** 2025-07-13

**Authors:** Bing Li, Yuanjun Tang, Zhanya Huang, Lijun Ma, Jiagui Song, Lixiang Xue

**Affiliations:** Cancer Center of Peking University Third Hospital, Beijing 100191, China; Department of Human Anatomy, Histology and Embryology, School of Basic Medicine, Ningxia Medical University, Yinchuan 750004, China; Cancer Center of Peking University Third Hospital, Beijing 100191, China; Peking Key Laboratory of Interdisciplinary Research in Gastrointestinal Oncology (BLGO), Peking University Third Hospital, Beijing 100191, China; Cancer Center of Peking University Third Hospital, Beijing 100191, China; Peking Key Laboratory of Interdisciplinary Research in Gastrointestinal Oncology (BLGO), Peking University Third Hospital, Beijing 100191, China; Cancer Center of Peking University Third Hospital, Beijing 100191, China; Department of Human Anatomy, Histology and Embryology, School of Basic Medicine, Ningxia Medical University, Yinchuan 750004, China; Cancer Center of Peking University Third Hospital, Beijing 100191, China; Department of Human Anatomy, Histology and Embryology, School of Basic Medicine, Ningxia Medical University, Yinchuan 750004, China; Center of Basic Medical Research, Institute of Medical Innovation and Research, Peking University Third Hospital, Beijing 100191, China; Cancer Center of Peking University Third Hospital, Beijing 100191, China; Department of Human Anatomy, Histology and Embryology, School of Basic Medicine, Ningxia Medical University, Yinchuan 750004, China; Center of Basic Medical Research, Institute of Medical Innovation and Research, Peking University Third Hospital, Beijing 100191, China

**Keywords:** organ-on-a-chip, microphysiological systems, organoids, disease modeling, animal trial alternatives, precision medicine

## Abstract

FDA issued guidance on 10 April 2025 to phase out animal trials in favor of organoids and organ-on-a-chip systems. This pivotal move was swiftly followed by the National Institutes of Health (NIH) on April 29th, when it inaugurated the Office of Research Innovation, Validation, and Application (ORIVA). The establishment of ORIVA aims to spearhead the advancement of human-centric organ-on-a-chip technologies, marking a major stride toward more accurate, ethical, and efficient research methods in the biomedical field. Compared to traditional 2D cell cultures and animal models, organ-on-a-chip systems enable precise control of hydrodynamic parameters and biomechanical microenvironments. This review systematically elaborates on applications of single-organ, multi-organ, and organoid-on-a-chip technologies in modeling complex diseases, host–microbiome interactions, inter-organ physiological networks, and quantitative prediction of pharmacokinetics, toxicity responses, and personalized therapies. Furthermore, the core challenges in translating these technologies to pharmaceutical development and clinical practice are critically analyzed. With interdisciplinary integration of materials engineering, biosensing, and artificial intelligence, organ-on-a-chip technologies are transcending the limitations of conventional preclinical research. Their strategic value as "patient surrogates" is poised to accelerate breakthroughs in precision medicine and rare disease treatments.

## Introduction

Traditional drug development relies on 2D/3D cell models and animal testing, yet their limitations impede clinical translation efficiency. Approximately 90% of drugs passing preclinical evaluations fail in clinical trials due to inaccurate human response predictions. Core challenges include interspecies discrepancies, static micro-environmental conditions, and insufficient cellular complexity. Conventional 2D/3D models lack vascular systems, dynamic mechanical cues, and multicellular interactions, limiting their ability to replicate organ functionality (Bale et al., 2014). Meanwhile, animal models face translational species barriers and prolonged experimental cycles (Soldatow et al., 2013). These predictive gaps escalate research and development costs and delay drug industrialization.

FDA issued guidance on 10 April 2025 to phase out animal trials in favor of organoids and organ-on-a-chip systems. This pivotal move was swiftly followed by the National Institutes of Health (NIH) on April 29th, when it inaugurated the Office of Research Innovation, Validation, and Application (ORIVA). The establishment of ORIVA aims to spearhead the advancement of human-centric organ-on-a-chip technologies, marking a major stride towards more accurate, ethical, and efficient research methods in the biomedical field (2025). This policy permits pharmaceutical companies to submit non-animal experimental data derived from organoids and organ-on-a-chip platforms as primary evidence for regulatory approval, prioritizing innovators adopting these advanced testing strategies. This initiative follows the implementation of the FDA Modernization Act 2.0 (2022) (Wadman, 2023) and FDA Modernization Act 3.0 (2024). Focused on reconstructing drug safety assessment frameworks, this reform marks a global transition toward “human-relevance” in pharmaceutical regulation.

This review systematically elaborates on applications of single-organ, multi-organ, and organoid-on-a-chip technologies in modeling complex diseases, host–microbiome interactions, multi-organ systemic physiological networks (e.g., gut–liver axis, blood–brain barrier [BBB]), and precise prediction of pharmacokinetics, toxicity responses as well as personalized therapies. In the meanwhile, the challenges are pinpointed and the perspectives are proposed.

## The syncretic convergence of organoid and organ-on-a-chip technological paradigms

Organoids are mini organ-like structures self-organized from pluripotent stem cells (PSCs) or adult stem cells under three-dimensional (3D) culture conditions *in vitro* (Clevers, 2016). By recapitulating spatiotemporal signaling pathways inherent to *in vivo* development, these constructs spontaneously form cellular compositions, spatial architectures, and functional properties remarkably akin to native organs, earning them the moniker “organs-in-a-dish” (Yan et al., 2023). Since the first successful generation of intestinal organoids in 2009 (Sato et al., 2009), this technology has expanded to model diverse organ systems, including the brain, liver, and kidneys (Park et al., 2023; Shek et al., 2021; Yousef Yengej et al., 2020). Unlike traditional 2D cell models, organoids preserve patient-specific genetic profiles and pathological phenotypes, demonstrating unique advantages in disease mechanism elucidation, personalized drug screening, and regenerative medicine (Lancaster and Knoblich, 2014). Current research prioritizes enhancing organoid vascularization, immune microenvironment reconstruction, and long-term culture stability to accelerate their translation into clinical applications such as precision medicine and organ replacement therapies (Rossi et al., 2018).

Organ-on-a-chip (OoC) is a bioinspired microfluidic platform that dynamically mimics the physiological microenvironment and functional units of human organs on a centimeter-scale chip by integrating living cells, biomaterials, and engineering design (Cao et al., 2023). This technology uses biocompatible materials like polydimethylsiloxane (PDMS) to create micron—scale channels which can precisely replicate biomechanical forces (e.g., fluid shear stress, cyclic mechanical stretching) and biochemical cues (e.g., oxygen levels, cytokine concentrations), including oxygen gradients and cytokine concentrations, thereby mimicking tissue architecture, metabolic functions, and pathological responses at the organ level. Since the pioneering development of “lung-on-a-chip” by Donald E. Ingber in 2010 (Huh et al., 2010), OoC systems have expanded to model diverse organs (e.g., heart, liver, BBB) and multi-organ interaction networks (Ingber, 2022). In contrast to conventional animal models, OoC platforms mitigate interspecies translational barriers by generating human-relevant pharmacological data on drug toxicity, metabolism, and pharmacokinetics. This capability marks a transformative advancement in preclinical research, leading to its formal designation as a new approach methodology (NAM) under modern regulatory frameworks, as endorsed by the U.S. FDA (2025). Current advancements focus on standardizing chip fabrication protocols, elucidating inter-organ communication mechanisms, and integrating AI-driven automated detection systems to accelerate applications in personalized medicine, environmental toxicology, and aerospace medicine (Koyilot et al., 2022; Leng et al., 2023).

Organoid-on-a-chip represent a next-generation biological model that combines the self-organizing properties of organoids with the engineering precision of organ-on-a-chip systems (Baptista et al., 2022). By embedding organoids into microfluidic platforms equipped with dynamic culture systems, this technology enhances organoids with physiological complexity through vascularization and immune cell recruitment, while preserving the genetic heterogeneity of patient-derived cells. The key breakthrough lies in integrating the inherent self-organizing capacity of organoids with the external engineering controls of OoC systems. This innovative combination addresses two critical limitations. First, conventional organoids often lack precisely controllable microenvironments. Second, traditional OoCs struggled to recapitulate cellular diversity. Organoid-on-a-chip is a subset or an advanced version of organ-on-a-chip. However, strictly speaking, traditional organ-on-a-chip systems (e.g., lung-on-a-chip, liver-on-a-chip) do not incorporate organoids, whereas organoid-on-a-chip represents a fusion of both technologies.

## Overview of organ-on-a-chip

### Technological evolution of organ-on-a-chip

**Figure 1.**
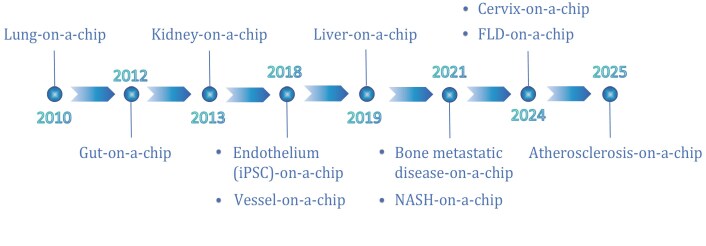
Timeline of organ-on-a-chip development.

A landmark in organ-on-a-chip (OoC) technology emerged in 2010 with the first functional human lung-on-a-chip developed by Donald Ingber’s team at Harvard’s Wyss Institute. By seeding pulmonary microvascular endothelial cells and alveolar epithelial cells into two adjacent channels of a microfluidic system, and utilizing a microchamber structure to introduce gases and cyclic stretching, they established a multifunctional microdevice replicating the structural, functional, and mechanical properties of living lung tissue. This system demonstrated the regulatory role of mechanical forces in pulmonary toxicity and function, marking a historic advancement in non-animal model applications for drug screening (Huh et al., 2010). In 2012, the inaugural intestine-on-a-chip combined human intestinal epithelial Caco-2 cells and microbial symbionts to recapitulate human intestinal physiology (Kim et al., 2012), followed by a kidney-on-a-chip was constructed for drug transport and nephrotoxicity assessment in 2013 (Jang et al., 2013). Subsequent developments included: a vascular-on-a-chip evaluating thrombosis mechanisms (2018) (Barrile et al., 2018); an iPSC-derived brain-on-a-chip replicating blood–brain barrier function (2018) (Sances et al., 2018); a blood–brain–barrier-on-a-chip enabling personalized medicine applications (2019) (Vatine et al., 2019); and bone-on-a-chip was developed to study osteogenic differentiation *in vitro*, coupled with optical imaging for non-destructive monitoring of cell viability, proliferation, and differentiation (2019) (Sheyn et al., 2019). Notable validation studies demonstrated liver-on-a-chip systems for cross-species drug hepatotoxicity assessment (Jang et al., 2019), alcoholic liver disease-on-a-chip for fibrosis research (2021) (Nawroth et al., 2021), and bone metastasis-on-a-chip investigating mechanical regulation of cancer invasion (Verbruggen et al., 2021). Recent advances include a fatty liver-on-a-chip with multi-omics validation (2024) (Perry et al., 2024), cervical-on-a-chip for host–microbiome interactions (Izadifar et al., 2024), and atherosclerosis-on-a-chip simulating plaque microenvironments (Hanford et al., 2025). For organoid integration, Toshiro Sato et al. first established a mouse intestinal organoid at Utrecht University in the Netherlands (Sato et al., 2009). While traditional organoids better replicate 3D tissue architecture than 2D cultures (Pampaloni et al., 2007), they exhibit significant limitations in simulating tissue-tissue interfaces and integrating dynamic mechanical forces (Ingber, 2006). Breakthroughs in microfluidic and 3D printing technologies now allow precise regulation of mechanical parameters (e.g., cyclic stretching, shear stress, and substrate stiffness) and biochemical gradients in the cellular microenvironment (Huh et al., 2010), with current research focusing on vascularization, standardization, and multi-organ integration. Figure 1 summarizes this developmental timeline.

### Current structural design and technical features of organ-on-a-chip systems

OoC platforms typically consist of culture chambers and microfluidic channels. The culture chambers provide three-dimensional growth spaces for cells or organoids, often fabricated from biocompatible materials such as PDMS or cycloolefin copolymer (COC)/cycloolefin polymer (COP). Chamber compartmentalization is achieved through either porous membrane (e.g., PDMS membranes) that enable paracellular substance exchange between upper and lower microchannels (Sharma et al., 2024) or membrane-free designs utilizing extracellular matrix (ECM) gels or micropillar arrays to segregate culture regions, thereby enhancing cell–cell interactions (Kosim et al., 2022). These chambers integrate microfluidic networks to regulate medium flow, mimicking *in vivo* blood circulation or tissue interstitial fluid exchange. Advanced systems incorporate pneumatically or mechanically actuated valves for dynamic perfusion control (Grant et al., 2021) or embed microelectrodes, cantilevers, or optical sensors for real-time monitoring of physiological parameters, such as pressure, dissolved oxygen levels, and electrical signals (Gallo et al., 2024; Harrell et al., 2024).

In terms of material selection, organ-on-a-chip platforms commonly utilize PDMS, COC/COP, PMMA (polymethyl methacrylate), or glass. PDMS offers flexibility and optical transparency but exhibits high small-molecule adsorption (Carius et al., 2024). COC/COP, characterized by high barrier properties and low adsorption, is preferred for drug metabolism studies (Olaizola-Rodrigo et al., 2024). PMMA provides high transparency for optical detection (e.g., Hesperos’ chip encapsulation), while glass is often employed as a base material for stable channel fabrication (Maschmeyer and Kakava, 2022). Advanced systems incorporate specialized materials such as ECM-mimicking hydrogels or rigid polymers. Hydrogels support cell adhesion and migration by replicating ECM properties, whereas rigid polymers address PDMS limitations in drug adsorption.

In terms of fluid control strategies, different organ-on-a-chip typically employ distinct driving mechanisms and fluid pathway designs. Driving mechanisms can be generally divided into three main types. The first type is pneumatic-driven systems; these systems make use of positive or negative pressure to operate microvalves. This allows for the precise simulation of physiological fluid parameters, such as shear stress and flow rate (Ishahak et al., 2020). The second type is gravity-driven systems; these systems depend on rocking motions or the force of gravity to perform perfusion. While they offer the advantage of easy operation, they have a limitation in achieving uniform shear stress (Busek et al., 2023). The third type is mechanical pump-driven systems (Komeya et al., 2016), such systems utilize external syringe or peristaltic pumps, making them well-suited for conducting high-throughput experiments.

Regarding the fluid pathway design, there are two main configurations. The first is single-organ perfusion, which uses independent culture units (Watson et al., 2017). This setup allows for the focused study of a single organ’s functions within a controlled environment. The second configuration is multi-organ cascades. In multi-organ cascades, interconnected microchannels play a crucial role (Liu et al., 2019). They link various organ models together, enabling the simulation of systemic metabolic interactions. For instance, it can effectively mimic the complex crosstalk that occurs between the liver, intestine, and kidney, providing valuable insights into how different organs interact and influence each other’s functions in the body (Koenig et al., 2022; Ronaldson-Bouchard et al., 2022).

In terms of analytical approaches, organ-on-a-chip systems enable the collection and analysis of diverse data while remaining compatible with traditional endpoint detection methods used in cell culture or clinical settings. The primary analytical methods include effluent analysis (such as metabolite secretion and exosome release) (Hanford et al., 2025), imaging analysis (such as confocal microscopy and multiphoton microscopy to observe cellular morphology, marker expression, and migration behaviors *in situ*) (Peel et al., 2019), and omics analysis (such as single-cell omics and proteomics) (Mozneb et al., 2024).

Organ-on-a-chip systems have emerged as transformative platforms for high-fidelity simulation of organ physiological and pathological processes through the integration of microfluidic technology, biomimetic materials, and dynamic regulation strategies. Current technological advancements are accelerating toward multi-organ integration, intelligent monitoring, and clinical translation, positioning these systems as potential cornerstone tools to replace animal testing and advance precision medicine.

The product features and key technical specifications of leading organ-on-a-chip platforms are summarized in Table 1.

**Table 1.** Comparison of leading organ-on-a-chip companies and technologies.

Company/ Country	Core products	Organ models	Key technologies	Fluid control	Materials/Design	Applications
**Emulate** **(USA)**	Chip S1/A1/R1	Liver, intestine, lung, kidney, brain	PDMS porous membrane separation, integrated mechanical stress (vacuum channels)	Pneumatic -driven (positive/ negative pressure)	PDMS (S1/A1), rigid polymer (R1)	Drug metabolism, toxicity evaluation, disease modeling
**CN Bio (UK)**	Dual-Organ Plate	Multi-organ interconnects (e.g., liver-intestine)	Open-access culture wells, programmable fluid parameters	Gas pressure-driven elastic diaphragm	Microfluidic channels + pneumatic valves	Drug development, toxicology studies
**TissUse** **(Germany)**	Chip2/Chip4 Series	Liver-kidney, liver-intestine (up to 4 organs)	Removable Transwell-like porous membrane chambers, PDMS microvalve sequencing	Pneumatic microvalve cycling perfusion	PDMS channels + glass substrate	Drug distribution /metabolism, multi-organ toxicity testing
**Beonchip** **(Spain)**	Be-Double flow (4 models)	Tumor, endothelial/epithelial barriers, immune models	COP/COC anti-adsorption material, dual-channel porous membrane separation	Syringe pump/peristaltic pump/rocking system	Cyclic olefin polymer (COP/COC)	Cell migration, pathogen–host interactions
**Mimetas** **(The Netherlands)**	OrganoPlate	High-throughput multi-organ models	Phase Guide technology (membrane-free), ECM gel-based cell distribution	Rocking perfusion (pump-free)	Glass base + polymer channels	Cell-cell interaction, migration studies
**Hesperos** **(USA)**	Human-on-a -Chip	Heart, liver, lung, brain (2–5 organs)	Pump-free gravity-driven perfusion, integrated microelectrodes /real-time sensors	Gravity-driven rocking perfusion	PMMA + PDMS + semiconductor sensors	Drug off-target toxicity, physiological modeling
**InSphero** **(Switzerland)**	Akura Flow Series	Organoid models (up to 12 organs)	Conical well design, specialized channel coatings for organoid stability	Microfluidic perfusion	Low-adhesion coating channels	Multi-organ drug testing, immune cell interactions
**Xilis** **(USA)**	MicroOrgano Sphere (MOS)	Tumor (preserved immune microenvironment)	Microfluidic droplet encapsulation, AI-driven drug prediction	Droplet technology	Gel droplets + PVDF membrane	Personalized medicine, tumor drug sensitivity testing
**Biomimx** **(Italy)**	Ubeat Platform	Heart, cartilage, intestine	Micro-pillar arrays for mechanical stress (stretch/compression)	Not specified	Elastic polymer material	Fibrosis modeling, mechanobiology studies
**AIM Biotech (Singapore)**	indeTx 3D Chip	Vascular, Neural, Cell Migration	Micro-pillar arrays + gas-permeable encapsulation, physiological oxygen gradients	Not specified	Optically transparent gas-permeable material	Angiogenesis, blood–brain barrier studies

## Single-organ chips

### Digestive system

#### Intestine-on-a-chip

##### Cell selection

The intestinal tract contains a variety of cell types, including epithelial cells, immune cells, and stromal cells. As a complex organ reliant on multicellular coordination, its chip model must be precisely tailored to align with distinct research objectives. The cell selection strategy for intestine-on-a-chip systems can be categorized into physiological models and pathological models (Tables 2 and 3).

**Table 2.** Physiological single-organ-on-a-chip models.

Organ	Model type	Cell type	Biomechanical system		References
			Flow-rate (μL/h)/Shear stress (dyn/cm^2^)	Mechanical stress	
Intestine	Drug efflux	Caco-2 cells + HUVECs	30 μL/h	10%, 0.15 Hz	(Carius et al., 2024)
	Drug absorption	Caco-2 cells (HTB-37)	30 μL/h, 0.02 dyn/cm^2^ + 125 μL/h	N/A	(Zhang et al., 2024)
	Drug absorption	Caco-2 cells (HTB-37)	30 μL/h, 0.02 dyn/cm² + 125 μL/h	N/A	(Gleeson et al., 2024)
	Intestinal barrier with crypt–villus axis	Intestinal epithelial cells (hiPSC) + mesenchymal cells (myofibroblasts, neurons); neuronal cells	0.067 mPa + 0.12–0.29 mPa + 1.7 mPa	N/A	(Moerkens et al., 2024)
	Biopsy derived organoids small intestine-on-a-chip	IEC + HIMECs	60 μL/h	10%, 0.2 Hz	(Kasendra et al., 2018)
	Microfluidics-based *in vitro* model	Caco-2 cells; CCD-18Co cells + primary CD4⁺ T cells + LGG	650 µL /min + 5 μL/min + 25 μL/min	N/A	(Shah et al., 2016)
	Inflammatory processes model	IEC (HIO) + monocyte-derived macrophages	121.2 μL/h	N/A	(Beaurivage et al., 2020)
	Culture of gut microbiome model	Caco-2; primary human ileal epithelial cells; Bacteroides fragilis + HIMECs	60 μL/h + 1 μL/min + 50 μL/min	10%, 0.15 Hz	(Jalili-Firoozinezhad et al., 2019)
Liver	Immune cell	Huh-7 + LSEC; PBMCs	0.05 Pa (0.5 dyn/cm^2^)	N/A	(Kennedy et al., 2024)
	Toxicology model	PHHs + sinusoidal endothelial cells; Kupffer cells; stellate cells	The physiological shear force of the hepatic sinusoids	N/A	(Ewing et al., 2025)
	Liver complex *in vitro* model	Hepatocytes (iPSC) + LSECs; Kupffer cells; stellate cells	0.1–1 mL/h + pulsatile flow	N/A	(Jadalannagari and Ewart, 2024)
	Metabolite analysis and pharmacokinetic evaluation	Primary human hepatocytes + Kupffer cells	1.0 µL/s	N/A	(Sarkar et al., 2015)
	Drug toxicities	Primary hepatocytes + LSECs; Kupffer cells; hepatic stellate cells	30 μL/h	N/A	(Jang et al., 2019)
	Modeling therapeutic antibody–small molecule drug-drug interactions	Primary human hepatocytes; Kupffer cells	1.0 µL/s	N/A	(Long et al., 2016)
Brain	Screening of BBB-crossing therapeutics	Cortical glutamatergic neurons (hiPSC); GABAergic neurons (hiPSC); PHA; microglia (hiPSC); PHP + iBMECs	30 μL/h (0.01 dyn/cm^2^)	N/A	(Chim et al., 2024)
	Drug blood–brain–barrier permeability	NHA; HBVP + hBMEC	80 μL/h + 30 μL/h	N/A	(Yang et al., 2024)
	Nanocarrier transport in blood–brain–barrier microvasculature	iPSC-Ecs; primary brain pericytes; astrocytes; HUVECs; HBMECs	N/A	N/A	(Lee et al., 2020)
	Blood–brain barrier	Primary human astrocytes; primary human brain pericytes + iBMECs	30 μL/h (0.01 dyn/cm^2^) + 3,600 μL/h (5 dyn/cm^2^)	N/A	(Vatine et al., 2019)
Heart	Heart-on-a-chip microphysiological system	hiPSCs; HGF + HUVECs	80 μL/h	N/A	(Liu et al., 2024)
	Drug cardiotoxicity	hiPSC-CMs + hiPSC-ECs	30 μL/h	N/A	(Mozneb et al., 2024)
	Application of 3D micro fibrous scaffolds in the heart	HUVECs; hiPSC-cardiomyocytes	5 µL/min + 50 µL/min	N/A	(Zhang et al., 2016)
Kidney	Specific morphogenesis and barrier function in human kidney-on-a-chip	Podocytes (hiPSC) + vascular endothelial cells (CD144⁺/CD31⁺)	1 µL/min + 4.09 µL/min	N/A	(Mou et al., 2024)
	iPSC reconstitutes kidney glomerular-capillary-wall function on a chip	hiPSC-podocytes + PH Glom ECs	60 μL/h (0.0007 dyn/cm²)	10%, 1 Hz	(Musah et al., 2017)
	Hipsc establishment of a glomerulus	Intermediate mesoderm cells (hiPSC) + HGMECs	N/A	10%, 1 Hz	(Musah et al., 2018)
	Physiological replication of the human glomerulus	hiPSC-IMD cells + GECs; GMCs	60 μL/h (0.0007 dyn/cm²)+ 60 μL/h (0.136 dyn/cm²)	N/A	(Pajoumshariati et al., 2023)
	Glomerular filtration barrier	hpPOD; hiPOD; hAKPC-P; hGEC	0.0117 Pa	N/A	(Petrosyan et al., 2019)
	Personalized glomerulus-on-a-chip engineered	ViECs + intermediate mesoderm cell	60 μL/h (0.017 dyn/cm²)+ 60 μL/h (0.0007 dyn/cm² )	10%, 0.4 Hz	(Roye et al., 2021)

For the “Cell type” column, “+” is used to distinguish cell types from different channels and “;” is used to distinguish cell populations within the same channel. For the “Biomechanical system” column under flow-rate (μL/h)/shear stress (dyn/cm²), “+” is used to distinguish flow rates (μL/h) and shear stresses (dyn/cm²) across distinct channels.

“HUVECs”: Human primary umbilical vein endothelial cells; “IECs”: Intestinal epithelial cells; “HIMECs”: Human intestinal microvascular endothelial cells; “LGG”: *Lactobacillus rhamnosus* GG; “HIOs”: Human intestinal organoids; “LSECs”: Liver sinusoidal endothelial cells; “PBMCs”: Peripheral blood mononuclear cells; “PHHs”: Primary human hepatocytes; “PHAs”: Primary human astrocytes; “PHPs”: Primary human pericytes; “iBMECs”: Induced brain microvascular endothelial cells; “NHAs”/“HAs”: Human astrocytes; “HBVPs”: Human brain vascular pericytes; “hBMECs”: Human brain microvascular endothelial cells; “HBMECs”: Human brain microvascular endothelial cells; “iBMECs”: Induced pluripotent stem cell (iPSC)-derived brain microvascular endotheliallike cells; “hiPSCs”: Human induced pluripotent stem cells; “HGFs”: Human gingival fibroblasts; “hiPSC-CMs”: Human induced pluripotent stem cell-derived cardiomyocytes; “hiPSC-ECs”: Human induced pluripotent stem cell-derived endothelial cells; “HGMECs”: Primary human glomerular microvascular endothelial cells; “hiPSC-IMDs”: Intermediate mesoderm cells derived from human induced pluripotent stem cells; “GECs”: Glomerular endothelial cells; “GMCs”: Glomerular mesangial cells; “hpPODs”: Primary podocytes; “hiPODs”: Immortalized podocytes; “hAKPC-Ps”: Amniotic fluid-derived podocytes; “hGECs”: Primary human glomerular endothelial cells; “ViECs”: Vascular endothelial cells.

**Table 3.** Pathological single-organ-on-a-chip models.

Organ	Model type	Cell type	Biomechanical system		References
			Flow-rate (μL/h)/Shear stress (dyn/cm^2^)	Mechanical stress	
Intestine	IBD	Colonic epithelial organoid cells (IBD) + fibroblast; hPBMC	60 μL/h	10%, 0.15 Hz	(Özkan et al., 2024)
	CDI (Clostridioides difficile Infection)	Caco-2 cells; HT29-MTX	30 μL/h	10%, 0.15 Hz	(Meza-Torres et al., 2025)
	Inflammatory bowel disease	HCoEpiC (human colonic epithelial cells)	60 μL/h	N/A	(Sontheimer-Phelps et al., 2020)
	Coxsackie B1 virus infection	Caco-2 cells	30 μL/h (0.02 dyn/cm^2^); 60 μL/h	10%, 0.15 Hz	(Villenave et al., 2017)
	Gastrointestinal infection of COVID-19	Caco-2 cells; HT-29 + HUVECs, circulating immune cells	200 μL/h; 50 μL/h	N/A	(Guo et al., 2021)
	Intestine radiation injury	Caco-2 cells + HUVECs	30 μL/h (0.02 dyn/cm²)	10%, 0.15 Hz	(Jalili-Firoozinezhad et al., 2018)
Lung	Influenza virus infection	PAEpiC (including type I and type II alveolar cells) + Macrophages (differentiated from CD14⁺HPMEC)	30 μL/h	N/A	(Man et al., 2024)
	SARS-CoV-2 infection	PAEpiC (containing AT1 and AT2 cells) + HLMVECs (H-6011)	30 μL/h	2%/5%, 0.2 HZ	(Suligoj et al., 2024)
	Mycobacterium fortuitum lung infection	HPAEpiC (AT1 and AT2 cells); Macrophages + HMVECs (Lonza CC-2527)	30 μL/h	5%, 0.2 Hz	(Ektnitphong et al., 2024)
	Pulmonary diving disease model	Primary human alveolar epithelial cells + primary human lung microvascular endothelial cells	30 μL/h	N/A	(Harrell et al., 2024)
Liver	Radiation injury	PHHs + LSECs	0.1–0.5 dyn/cm²	N/A	(Martello et al., 2024)
	Metabolic dysfunction-associated steatotic liver disease; non-alcoholic fatty liver disease	PHHs + NPCs (including LSECs, hepatic stellate cells, Kupffer cells)	N/A	N/A	(Perry et al., 2024)
	Nonalcoholic steatohepatitis	LSEC + HCs; KCs; HSCs	N/A	N/A	(Freag et al., 2021)
Brain	Humans neuroinflammation model	Glutamatergic neurons (iPSC); GABAergic neurons (iPSC); PHA; human microglia cell line; PHP + iBMECs	60 μL/h	N/A	(Pediaditakis et al., 2022)
	Blood–brain barrier disruption model	Dopaminergic (iPSC); neurons; PHA; PHM; PHP + HBMECs (iPSC)	30 μL/h 60 μL/h	N/A	(Pediaditakis et al., 2021)
	Inflammatory disruption of the blood–brain barrier	Primary human astrocytes; primary human brain vascular pericytes; neurons (hiPSC); co-differentiating astrocytes + hBMVEC	2 µL/min	N/A	(Brown et al., 2016)
	Fungal brain infection	Ecs; PCs; Huh-7; NSCs	0–6 dyn/cm²	N/A	(Kim et al., 2021)
Kidney	Renal injury in human cardiorenal syndrome	hRPTECs	N/A	N/A	(Chatterjee et al., 2023)
	Phenocopies low syndrome and dent II disease tubulopathy	HK-2/HK-2	N/A	N/A	(Naik et al., 2021)
	Virus-related renal dysfunctions	MDCK	5 × 10^−4^ Pa	N/A	(Wang et al., 2019)
	Hypertensive nephropathy	GEnCs + MPC-5	5–15 μL/min (0.001–0.003 dyn/cm²)	N/A	(Zhou et al., 2016)

For the “Cell type” column, “+” is used to distinguish cell types from different channels and “;” is used to distinguish cell populations within the same channel. For the “Biomechanical system” column under flow-rate (μL/h)/shear stress (dyn/cm²), “+” is used to distinguish flow rates (μL/h) and shear stresses (dyn/cm²) across distinct channels.

“hPBMCs”: Human peripheral blood mononuclear cells; “HCoEpiCs”: Human colonic epithelial cells; “HUVECs”: Human primary umbilical vein endothelial cells; “PAEpiCs”: Primary lung alveolar epithelial cells; “HPMECs”: Human pulmonary microvascular endothelial cells; “HLMVECs”: Human lung microvascular endothelial Cells; “HPAEpiCs”: Human primary lung alveolar epithelial cells; “HMVECs”: Human microvascular endothelial cells; “PHHs”: primary human hepatocytes; “LSECs”: Liver sinusoidal endothelial cells; “NPCs”: Non-parenchymal cells; “HCs”: Hepatocytes; “KCs”: Kupffer cells; “HSCs”: Hepatic stellate cells; “iBMECs”: Induced pluripotent stem cell (iPSC)-derived brain microvascular endothelial like cells; “PHAs”: Primary human astrocytes; “PHM”: Human primary microglia; “PHPs”: Primary human pericytes; “HBMECs”: Human brain microvascular endothelial cells; “ hBMVEC”: Human brain microvascular endothelial cells; “Ecs”: Endothelial cells; “PCs”: Pericytes; “NSCs”: Neural stem cells; “hRPTECs”: Human renal proximal tubular epithelial cells; “MDCK”: Madin darby canine kidney cells; “GEnCs”: mice glomerular endothelial cell; “MPC-5”: Mouse podocyte cell line 5.

Physiological models are typically constructed using human colon adenocarcinoma cell lines, such as Caco-2 (Guo et al., 2021; Jalili-Firoozinezhad et al., 2018, 2019; Villenave et al., 2017), induced pluripotent stem cells (iPSCs), organoids (Kasendra et al., 2018), or vascularized-enhanced models co-cultured with primary human umbilical vein endothelial cells (HUVECs) (Carius et al., 2024; Guo et al., 2021; Kasendra et al., 2018). For organoid-on-a-chip, organoids were usually differentiated from human iPSCs (hiPSCs) or derived from intestinal crypts isolated from patient biopsies (Sontheimer-Phelps et al., 2020), and dissociated into single cells for chip inoculation.

Pathological models can be constructed either by directly using disease-specific cell lines or by introducing exogenous pathogenic microbiota/engineered aberrant cells into existing physiological systems, thereby establishing disease phenotypes through infection or experimental induction. For inflammatory bowel disease (IBD) models, IBD patient-derived epithelial cells with fibroblasts, combined with hormonal stimulation (mimicking estrogen fluctuations) or carcinogen exposure (inducing mutations) were used (Beaurivage et al., 2020; Özkan et al., 2024). For infection or gastrointestinal microbiome models, disease can be constructed via infection or induction (Guo et al., 2021; Meza-Torres et al., 2025). For example, Pranjul Shah’s team cultured *Lactobacillus rhamnosus* GG (LGG) either alone or in co-culture with *Bacteroides caccae* (an obligate anaerobe) within microbial microchambers under anoxic conditions to establish gastrointestinal microbiome models, subsequently investigating the relationships between gut microbiota and human health/disease (Shah et al., 2016).

##### Culture system

The biomimetic design of intestine-on-a-chip requires balancing structural stability and physiological dynamics. Key experimental parameters encompass porous membrane engineering with ECM modifications (commonly utilizing Matrigel or collagen), precise fluidic dynamics control systems, and biomechanical stimulation through cyclic mechanical stretching protocols (Tables 2 and 3). A well-designed flow rate not only promotes cell adhesion on chips but also simulates the shear forces experienced by intestinal cells in physiological environments, thereby enhancing cell differentiation and the formation of specific physiological structures (Moerkens et al., 2024; Zhang et al., 2024). In addition to fluid flow, intestine-on-a-chip systems often incorporate mechanical stretching to mimic rhythmic peristaltic movements. Current stretching parameters for intestine-on-a-chip systems are generally set at 10% strain with a 0.15 Hz frequency to simulate peristaltic motion (Carius et al., 2024; Meza-Torres et al., 2025). However, specific parameters for specialized regions such as the ileocecal junction remain undefined. Further introduction and validation of these parameters are required based on physiological data from corresponding anatomical areas.

##### Translational applications

Intestine-on-a-chip systems are widely applied in the fields ranging from basic research to drug development. These include developing stem cell self-organization systems (Moerkens et al., 2024), modeling diseases with host–microbiome interactions in pathological contexts (Özkan et al., 2024), evaluating drug permeability in physiological conditions (Gleeson et al., 2024), and constructing drug absorption models.

#### Liver-on-a-chip

##### Cell selection

As the core metabolic and detoxification organ in the human body, the liver’s functions are critically simulated *in vitro* through biomimetic models, which hold significant importance for drug toxicity evaluation and disease mechanism research. The liver-on-a-chip reconstructs the hepatic microenvironment via a multi-cellular co-culture system, with cell selection strategies categorized into physiological models and pathological models.

Physiological models typically seed primary human hepatocytes (PHHs) and liver sinusoidal endothelial cells (LSECs) (Kennedy et al., 2024). To enhance the physiological relevance of the model, Kupffer cells and hepatic stellate cells are often incorporated to establish a multi-cellular collaborative system (Jadalannagari and Ewart, 2024; Kennedy et al., 2024).

Pathological models demonstrate diversified technical approaches. Direct seeding of tumor cell lines is a common strategy (Kennedy et al., 2024). Another strategy involves inducing pathological phenotypes through microenvironmental interventions (Ewing et al., 2025; Freag et al., 2021). For instance, a metabolic dysfunction-associated steatotic liver disease (MASLD) model can be established by treating a four-cell co-culture system (formed after culturing adult PHHs with non-parenchymal cells—Kupffer cells, hepatic stellate cells, and liver sinusoidal endothelial cells) with high concentrations of free fatty acids (oleic acid/palmitic acid) (Perry et al., 2024). Moreover, X-ray irradiation was used to construct a radiation-induced liver injury model (Martello et al., 2024). These models mimic injury patterns and functional abnormalities characteristic of inflammatory responses, thereby replicating the liver microenvironment under inflammatory conditions through these simulated injury patterns (Sarkar et al., 2015).

##### Culture systems

Liver-on-a-chip generally employs porous PDMS membranes modification with ECM (collagen and fibronectin are usually used) to to enhance hydrophilicity and cell adhesion (Jadalannagari and Ewart, 2024; Martello et al., 2024). In the process of constructing the *in vitro* model of the liver-on-a-chip, mechanical stress are usually excluded. This is mainly because the magnitude of mechanical deformation of the organ in the physiological state is considered to be at a negligible level. As for the setting of hydrodynamic parameters, precise regulation is required according to the anatomical region targeted for bionics (e.g., simulating the physiological shear stress environment of the liver sinusoid microcirculation or the characteristics of pulsatile hemodynamics) (Kennedy et al., 2024). It is worth noting that the introduction of such hydrodynamic stimuli should be carried out gradually only after the hepatic parenchymal cells and non-parenchymal cells have completed adhesion to the wall and established a stable three-dimensional tissue structure, so as to ensure the biological relevance of the functional construction (Perry et al., 2024; Sarkar et al., 2015).

##### Translational applications

The liver-on-a-chip finds extensive use in a diverse range of applications. It is employed to accurately assess drug toxicity, create models of metabolic liver diseases, replicate tumor immune microenvironments, explore the mechanisms of liver injury and investigate multicellular interactions. FDA-certified Emulate’s organ-on-a-chip platform to evaluate drug toxicity, its predictive efficacy for drug-induced liver injury (DILI) has been thoroughly validated (Jadalannagari and Ewart, 2024). The liver-on-a-chip assesses a drug’s hepatotoxicity risk compared to similar drugs by analyzing liver injury potential, determining whether candidates pose lower, equivalent, or higher risk than existing therapies. The Emulate human liver-on-a-chip detected 87% of clinically significant DILI-causing drugs that passed animal tests but caused fatal liver toxicity in humans. With 100% specificity in excluding non-toxic compounds, it demonstrates reliable safety profiling capability for drug development (Ewart et al., 2022). This system successfully recapitulates the recruitment process of immune cells within the hepatic microenvironment, offering an innovative tool for evaluating hepatotoxicity of immune checkpoint inhibitors (Kennedy et al., 2024). Liver-on-a-chip exhibits multifaceted advantages over traditional models. It reconstructs a three-dimensional complex microenvironment through co-culture system and the use of tissue-specific ECM provides *in vivo*-like growth support, enabling more authentic simulation of human liver physiology. In contrast, traditional chips often suffer from limitations such as single-cell-type systems, lack of physiological fluid simulation, generic matrix environments, lower predictive accuracy, restricted detectable phenotypes, inflexible parameter adjustment, and limited clinical relevance or applicability, making them unable to match the research and application efficacy of the liver-on-a-chip.

### Respiratory system

#### Lung-on-a-chip

##### Cell selection

The cell selection strategy for lung physiological chips is usually as follows: primary human alveolar epithelial cells (including type Ⅰ and type Ⅱ alveolar cells) are cultured at the air-liquid interface (ALI) in the top channel, while primary human lung microvascular endothelial cells (HMVECs) are cultured in the bottom channel, so as to simulate the vascular perfusion process (Ektnitphong et al., 2024; Harrell et al., 2024; Man et al., 2024; Suligoj et al., 2024). Pathological chips are based on the completion of lung physiological chip construction. By introducing pathogenic factors such as bacteria and viruses (Ektnitphong et al., 2024; Man et al., 2024; Suligoj et al., 2024), macrophages can be simultaneously implanted (Ektnitphong et al., 2024; Man et al., 2024), or immune cells derived from whole blood can be directly used (Harrell et al., 2024) to simulate the alveolar microenvironment under pathological conditions.

##### Culture system

Under physiological conditions, lung tissue undergoes continuous rhythmic expansion and contraction during breathing, accompanied by gas exchange in the alveoli. To simulate these physiological processes in lung-on-a-chip design, it is essential to replicate the ALI and mechanical stretching caused by respiratory movements. The porous PDMS membrane between the two channels separates the compartments, allowing gas exchange and cell adhesion. ECM coating typically employs a combination of collagen IV, fibronectin, and laminin (Ektnitphong et al., 2024; Harrell et al., 2024; Suligoj et al., 2024).

For fluid flow regulation, both channels initially receive continuous medium perfusion at 30 μL/h. However, fluid flow in the top channel is stopped during ALI establishment to mimic the physiological alveolar environment, enabling better epithelial cell differentiation at the air-liquid interface for research purposes (Ektnitphong et al., 2024; Man et al., 2024; Suligoj et al., 2024). The flow rate and medium composition in the lower channel are adjusted according to experimental requirements. To simulate the rhythmic breathing motion of lung tissue, mechanical stretching parameters are typically set to 5% strain at 0.2 Hz (Ektnitphong et al., 2024; Suligoj et al., 2024).

##### Translational applications

Lung-on-a-chip systems are generally used for pathological research, with in-depth exploration of disease mechanisms through various functional verification experiments. For example, virus infection and replication detection immunofluorescence staining are used to detect viral nucleocapsid proteins, and focus formation assays are used to quantify viral titers, confirming that SARS-CoV-2 can successfully infect epithelial cells in the chip and replicate. The lung-on-a-chip overcomes traditional model limitations in physiological relevance and mechanistic dissection through its biomimetic structural design (multicellular co-culture, ALI, dynamic fluidics), high-fidelity pathological models (infected/non-infected scenarios), and multidimensional functional validation capabilities. Its core advantage lies in simulating the dynamic characteristics of the human alveolar microenvironment, making it particularly suitable for studying host–pathogen interactions, immunopathological mechanisms, and drug screening. It provides a revolutionary tool for basic research and clinical translation of lung diseases such as influenza, COVID-19, mycobacterial infections, and decompression sickness.

### Nervous system

#### Brain-on-a-chip

##### Cell selection

Brain-on-a-chip systems primarily focus on constructing BBB models. The BBB, formed by brain capillary walls and neuroglial cells, acts as a selective barrier separating plasma from brain cells. Accordingly, the cells include the brain and vascular compartments. Brain compartment includes co-culture of cortical neurons, astrocytes, pericytes, microglia (Chim et al., 2024; Pediaditakis et al., 2021, 2022) or dopaminergic neurons to simulate the substantia nigra region (Pediaditakis et al., 2021), while the vascular compartment used either iPSC-derived brain microvascular endothelial-like cells (iBMECs) (Chim et al., 2024; Pediaditakis et al., 2021, 2022) or primary human brain microvascular endothelial cells.

##### Culture system

The 50 μm-thick PDMS porous membrane was often used to separate different channels, and the membrane was usually coated with collagen IV, fibronectin, and laminin. Current standardized protocols for brain organ-on-a-chip systems universally adopt a two-phase flow rate control strategy: Phase I involves static culture (1–2 days), promoting cell adhesion and early interactions through fluid-free quiescent conditions (Chim et al., 2024); Phase II implements dynamic perfusion via microfluidic systems to mimic physiological shear stress (0.01 dyn/cm²) separately through cerebral and vascular channels, which is maintained for 5–14 days to support tissue maturation (Yang et al., 2024). In BBB model construction, distinct strategies emerge across research groups (Lee et al., 2020).

##### Translational applications

Current research on brain-on-a-chip systems focuses on drug screening and BBB permeability assessment (Chim et al., 2024). This systematic validation framework ensures the reliability of BBB models for pathological studies (e.g., neuroinflammation, and α-synucleinopathies) and translational applications (Brown et al., 2016; Kim et al., 2021). Antibodies or viral vectors are introduced into the vascular channel, and their penetration into the brain channel is quantified. Similar methods are applied to measure drug penetration and calculate parameters such as the brain/blood concentration ratio (K_p_) and apparent permeability coefficient (P_app_) to validate drug permeability and toxicity. The advantages extend beyond simulating neurovascular unit interactions in basic research to accelerating central nervous system (CNS) drug development. Future integration with patient-specific iPSCs holds promise for advancing personalized medicine and precision therapies.

### Cardiovascular system

#### Heart-on-a-chip

##### Cell selection

hiPSC-derived cardiomyocytes (hiPSC-CMs), hiPSC-derived vascular endothelial cells (hiPSC-ECs) or coculture with human gingival fibroblasts (HGFs) and human umbilical vein endothelial cells (HUVECs) were often used in heart-on-a-chip.

##### Culture system

Matrigel or gelatin was used in the membrane coating. Fluid flow (30–80 μL/h) (Liu et al., 2024) and mechanical stretching (frequency of 0.4 Hz, deformation of 10%) were often applied (Mozneb et al., 2024; Zhang et al., 2016).

##### Translational application

Heart-on-a-chip models is usually used to evaluate drug cardiotoxicity. Its advantages lie in enabling multicellular co-culture (e.g., cardiomyocytes, endothelial cells, and fibroblasts), accurately mimicking drug toxicity and physiological responses across multiple cell lineages, and surpassing the limitations of traditional two-dimensional culture. This provides a highly biomimetic and quantifiable cross-species model for cardiovascular drug screening and pathological mechanism research, providing dual value by reducing animal use and enhancing clinical translation potential.

### Urinary and reproductive system

#### Kidney-on-a-chip

##### Cell selection

Most kidney-on-a-chip models simulate glomerular structures, while a minority replicate renal tubular architectures. In glomerulus-mimicking models, chips are typically seeded with hiPSC-differentiated podocytes to simulate the urinary compartment (Mou et al., 2024; Musah et al., 2017, 2018; Pajoumshariati et al., 2023) and endothelial cells to represent the vascular compartment. Endothelial cell sources include hiPSC-derived vascular endothelial cells or primary human endothelial cells (Mou et al., 2024; Musah et al., 2017; Naik et al., 2021). Beyond human-derived cells, the glomerulus-on-a-chip (GC) model developed by Mengying Zhou’s team offers an alternative approach. They constructed the GC by seeding mouse glomerular endothelial cells (GEnCs) in the upper layer, and mouse podocytes (MPC-5) in the lower layer of a microfluidic chip, successfully mimicking glomerular structure and functionality. This methodology demonstrates the feasibility of employing non-human cells as viable alternatives for organotypic models (Zhou et al., 2016).

##### Culture system

Most kidney-on-a-chip models use PDMS membranes (7 μm pore size). However, Xingrui Mou’s team developed electrospun silk fibroin (SF) membranes with a thickness of 3.5 μm, which is an order of magnitude thinner than traditional PDMS membranes. These ultrathin membranes were employed to study tissue-specific morphogenesis and barrier functions mediated by membranes in human kidney-on-a-chip models (Chatterjee et al., 2023). For ECM selection, collagen I and Laminin-511 are commonly used to coat the porous membranes (Musah et al., 2017, 2018; Naik et al., 2021). Further investigation into the physical parameters generated by this system (e.g., shear stress profiles) could potentially delineate critical biomechanical requirements for proximal tubule self-organization.

While flow rate details are rarely specified in the literature, mechanical stimulation parameters are described. For instance, 10% cyclic stretching (1 Hz) is applied to mimic the pulsatile mechanical stimuli of glomerular capillaries.

##### Translational application

In glomerulus-on-a-chip construction, most research validate glomerulus-like structural and functional characteristics, particularly the physiological barrier function (Pajoumshariati et al., 2023). The kidney-on-a-chip platform has emerged as a transformative tool in three key biomedical applications: (i) preclinical nephrotoxicity assessment through quantitative evaluation of chemotherapy-induced glomerular barrier dysfunction, preclinical drug safety profiling, animal-free prediction of drug-induced proteinuria, and accelerated therapeutic development for nephropathies (Musah et al., 2017; Wang et al., 2019); (ii) biomaterial innovation by demonstrating silkworm fibroin (SF) membranes with tunable mechanical properties and biodegradability as novel vascularized organ engineering substrates (Chatterjee et al., 2023); and (iii) CRISPR-based genetic modeling of hereditary kidney disorders including congenital nephrotic syndrome, enabling mechanistic interrogation of disease pathogenesis through precision medicine approaches.

## Multi-organ-on-a-chip systems

In recent years, microphysiological systems (MPS, also known as organ-on-a-chip systems), particularly multi-organ-on-a-chip platforms, have demonstrated significant advancements. These revolutionary *in vitro* tools, widely applied in drug development and disease modeling, replicate human inter-organ physiological interactions, thereby substantially improving the physiological relevance and predictive capacity of biomedical research.

### Application of multi-organ-on-a-chip systems in disease modeling

In the study of tumor metastasis and treatment, Aleksander Skardal’s team developed a “metastasis-on-a-chip” (MOC) system to simulate the tumor metastatic microenvironment using intestinal and liver constructs containing tumor foci. Multiple cell types were cultured and assembled into the MOC device, enabling real-time tracking of fluorescently labeled tumor cell migration via microscopy. Post-culture, immunohistochemistry was used to detect cell marker expression. By chemically modulating hydrogel mechanical properties or applying chemotherapeutic drugs, the invasive migratory behavior of tumor cells could be regulated. This validated MOC system demonstrates the ability to mimic tumor metastasis, study tumor cell behavior, and evaluate drug efficacy (Skardal et al., 2016). This model provides a critical tool for cancer research and anti-cancer drug development. In addition to its application in modeling cancer metastasis with MOC technology, research in diabetes modeling has also made significant progress. Sophie Bauer’s team developed a two-organ-on-a-chip (2-OoC) microfluidic model. This innovative model is designed to co-culture human islet microtissues and liver spheroids, effectively mimicking the functional coupling between human pancreatic islets and the liver in glucose regulation. After functional and morphological characterization, the islet microtissues and liver spheroids were cultured in the 2-OC system under insulin-free medium for 15 days in both monoculture and co-culture conditions. An *in vitro* glucose tolerance test (GTT) was conducted, during which glucose and insulin levels were measured at various time points. This approach enabled a comprehensive assessment of the interactions between different organs. The model successfully replicated the insulin-glucose feedback regulatory loop. Results demonstrated that islet microtissues released insulin under high-glucose conditions, which subsequently stimulated glucose uptake by liver spheroids, forming a functional regulatory circuit (Bauer et al., 2018). This model lays a foundation for studying diabetes pathophysiology and developing novel therapeutic strategies. Moreover, in combined skin and tumor studies, multi-organ-on-a-chip systems were utilized to evaluate drug efficacy and skin side effects in anti-epidermal growth factor receptor (EGFR) cancer therapy. For example, Juliane Hübner’s team developed a “safety-efficacy” testing approach by co-culturing human lung cancer microtissues and full-thickness skin equivalents to simultaneously assess the tumor-suppressive effects and skin toxicity of the anti-EGFR antibody cetuximab. Results showed that cetuximab not only inhibited tumor cell proliferation but also significantly impacted the renewal of basal skin layer cells and the secretion of related cytokines (Hubner et al., 2018). This model provides a novel strategy for evaluating the therapeutic index of anti-cancer drugs, with potential applicability to melanoma by adapting the platform to incorporate melanoma-specific tumor models.

### Application of multi-organ-on-a-chip systems in drug development

Firstly, in predicting drug efficacy and safety, multi-organ-on-a-chip systems play a pivotal role. For example, integrated multi-organ systems combining liver, heart, and skeletal muscle have been used to evaluate the efficacy and cardiotoxicity of anti-cancer drugs. Studies revealed that isolated tumor cells and cardiac tissues showed drug responses inconsistent with clinical data, whereas tumor and cardiac tissues cultured in integrated platforms more accurately simulated clinical scenarios (Oleaga et al., 2016). Similarly, liver-kidney integrated models were employed to study the nephrotoxicity mechanisms of aristolochic acid, demonstrating that liver-derived metabolites significantly contribute to renal toxicity (Chang et al., 2017). Multi-organ-on-a-chip systems also excel in multi-organ toxicity assessment. For instance, a system integrating heart, liver, skeletal muscle, and neurons was used to evaluate drug toxicity across multiple organs. Testing drugs with known side effects showed that the results derived from this system aligned with published human and animal data, confirming its reliability in predicting drug toxicity (Oleaga et al., 2016). Additionally, liver-skin integrated models validated their stability and reproducibility in long-term substance testing for drug safety evaluation. In drug metabolism and pharmacokinetic (PK) studies, multi-organ-on-a-chip systems are equally impactful. For example, intestine-liver integrated models were used to study drug absorption, metabolism, and excretion. By simulating the flow of drugs from the intestine to the liver, researchers quantified metabolic rates and clearance, providing critical data for early-stage drug development (Tsamandouras et al., 2017). Multi-organ-on-a-chip systems also show promise in reproductive toxicity assessment. For example, Y. Baert’s team developed a liver-testis integrated model using human-derived cells to construct testis organoids and liver spheroids. These were cultured in separate compartments of a multi-organ-on-a-chip system under distinct media for one week, followed by cyclophosphamide treatment. Metrics such as metabolic activity and cell viability were monitored, alongside immunohistochemical and real-time quantitative PCR analyses. Results demonstrated that liver metabolites significantly amplified cyclophosphamide’s toxicity to testis organoids, shedding light on mechanisms of reproductive toxicity (Baert et al., 2020).

### Application of multi-organ-on-a-chip systems in studying inter-organ interactions in physiological and pathological conditions

Multi-organ-on-a-chip systems can be employed to investigate metabolic coupling between endothelial and neuronal cells within the neurovascular unit (NVU). By integrating a BBB-on-a-chip with a brain-on-a-chip, researchers observed that fluidic coupling regulates cellular phenotypes in the NVU system. For instance, vesicular transport processes in vascular endothelial cells were downregulated, while cytoskeletal functional processes dominated in the brain-on-a-chip. Additionally, methamphetamine (Meth) was found to induce transient BBB opening, with more pronounced effects on BBB permeability (Maoz et al., 2018). The physiological and pathological crosstalk between intestinal and hepatic systems has been systematically investigated through multi-organ-on-a-chip platforms. Mechanistic studies demonstrate that fibroblast growth factor 19 (FGF19) exerts negative feedback regulation on hepatic bile acid synthesis via suppression of cytochrome P450 family 7 subfamily A member 1 (CYP7A1) (Chen et al., 2017). Multi-organ-on-a-chip systems are further utilized to study immune cell–organ interactions. For example, a system integrating THP-1 cells (a monocyte/macrophage lineage) with functional immune components was developed to monitor complex cellular behaviors induced by drug treatments (Sasserath et al., 2020). Studies demonstrated that THP-1 cells differentiated into M2 macrophages within damaged myocardial tissues, highlighting the system’s capacity to simulate dynamic interactions between immune cells and organs.

### Advantages and future directions of multi-organ-on-a-chip systems

Multi-organ-on-a-chip technologies offer significant advantages over traditional *in vitro* culture systems and animal models in the following four aspects: (i) High physiological relevance: Multi-organ-on-a-chip systems can mimic complex physiological and pathological processes in the human body, providing experimental models that closely resemble human conditions. (ii) Multifunctionality: By integrating multiple organs, these chips enable simultaneous study of diverse diseases and drug responses, significantly enhancing research efficiency. (iii) Cost-effectiveness and efficiency: They reduce reliance on animal experiments, lower research costs, and make the rapid acquisition of high-content experimental data possible. (iv) Quantitative analysis capability: Multi-organ-on-a-chip systems support quantitative pharmacokinetic and toxicity assessments, delivering precise data for drug development (Edington et al., 2018; Herland et al., 2020; Tsamandouras et al., 2017; Vernetti et al., 2017).

Despite these advancements, future development of multi-organ-on-a-chip systems still faces challenges: (i) Model complexity: Increasing the number of integrated organs elevates system complexity, complicating operational workflows and data analysis. (ii) Standardization issues: A lack of unified protocols for chip design and experimental procedures necessitates further optimization and standardization. (iii) Long-term functional stability: Maintaining functional stability of multi-organ systems during prolonged cultivation remains a critical technical hurdle to address.

## Summary and future perspectives

Modern biomedical research and drug development have long relied on two-dimensional cell cultures and animal models. However, these traditional models exhibit significant limitations in replicating the complexity of human physiological environments and predicting clinical responses. Statistics indicate that over 90% of drugs entering clinical trials fail due to inefficacy in reproducing animal study outcomes or unforeseen toxicities. This critical challenge has driven the emergence of OoC technologies, representing the third generation of *in vitro* models. By integrating microfluidics with tissue engineering, OoC systems construct dynamic MPS with organ-level functionality, offering a revolutionary platform for precision medicine and personalized therapies (Ingber, 2022).

Recent advancements in OoC technology have been driven by breakthroughs in two core areas. First, microfluidic technology enables precise control of cellular microenvironments, including fluid shear stress, mechanical forces, three-dimensional architecture, and spatiotemporal regulation of multicellular co-culture. This allows highly accurate recreation of physiological and pathological organ microenvironments. Second, organoid technology matures, allowing stem cells or primary cells to self-organize into organ-specific functional structures. These organoids provide biologically relevant units that closely mimic *in vivo* tissue complexity for integration into OoC systems.

The synergy between these technologies has given rise to organoid-on-a-chip systems, a novel subset of OoC platforms. By combining microfluidic systems with dynamic culture and phenotypic modulation of organoids, these systems significantly enhance the physiological relevance and functional durability of *in vitro* models (Peck et al., 2020).

Current advancements in OoC technology have evolved from single-organ simulations to multi-organ interaction systems. For example, gut-liver-on-a-chip simulates first-pass metabolism effects by circulating culture media, while BBB-neural-on-a-chip systems investigate the neurotoxicity mechanisms of nanoparticles. Concurrently, this technology is driving innovations in personalized medicine: disease model-on-a-chip derived from patient-specific iPSCs has demonstrated unique advantages in studying rare genetic disorders such as hereditary heart diseases and cystic fibrosis.

In terms of industrial translation, a landmark regulatory advancement has been the establishment of standardized “body-on-a-chip” platforms for drug evaluation, formally recognizing organ-on-a-chip (OoC) data as valid components of preclinical assessment frameworks. This represents a critical milestone in the regulatory acceptance of MPS (Peck et al., 2020).

Despite showcasing transformative potential in biomedical research, the large-scale industrial implementation of organ-on-a-chip is hindered by five fundamental hurdles: standardization constraints, system integration intricacies, and industrialization cost obstacles. First, variability in data reproducibility across chips struggles to meet the stringent requirements of good laboratory practices (GLP) for preclinical data—a critical issue for multinational pharmaceutical companies collaborating across multi-lab studies. Second, while multi-organ coupling (e.g., liver-kidney-intestine-on-a-chip systems) enables simulation of systemic toxicity effects, spatiotemporal discrepancies in inter-organ substance transfer efficiency (e.g., first-pass metabolism rate variations up to 30%) significantly constrain accurate modeling of complex toxicity mechanisms. Thirdly, high cost and manual operation cycles spanning struggle to meet the stringent throughput and cost requirements of high-throughput screening. These hurdles highlight the need for standardized protocols, modular system designs, and cost-reduction strategies to bridge the gap between experimental innovation and industrial scalability. Fourthly, cross-scale biological process simulation, replicating dynamic coupling across molecular-cellular-tissue-organ interfaces remains unresolved. Finally, due to engineering limitations in vascular networks and industrial—scale manufacturing gaps, the absence of ISO-standardized GMP production lines and automated detection platforms for organ-on-a-chip forces reliance on manual processes, forming efficiency bottlenecks. In the future, advancements in materials science, including 3D bioprinted dynamic scaffolds, bioinformatics, and nanoscale sensor integration, could overcome these bottlenecks. This would revolutionize biomedical research, shifting the paradigm from “animal experiment alternatives” to “human physiology reconstruction”.
